# Hedgehog proteins and parathyroid hormone‐related protein are involved in intervertebral disc maturation, degeneration, and calcification

**DOI:** 10.1002/jsp2.1071

**Published:** 2019-11-19

**Authors:** Frances C. Bach, Kim M. de Rooij, Frank M. Riemers, Joseph W. Snuggs, Willem A. M. de Jong, Ying Zhang, Laura B. Creemers, Danny Chan, Christine Le Maitre, Marianna A. Tryfonidou

**Affiliations:** ^1^ Department of Clinical Sciences of Companion Animals, Faculty of Veterinary Medicine Utrecht University Utrecht The Netherlands; ^2^ Biomolecular Sciences Research Centre, Sheffield Hallam University Sheffield UK; ^3^ School of Biomedical Sciences The University of Hong Kong Pokfulam Hong Kong; ^4^ Department of Orthopaedics University Medical Center Utrecht Utrecht The Netherlands

**Keywords:** calcification, IHH, IVD, PTHrP, SHH

## Abstract

Parathyroid hormone‐related protein (PTHrP) and hedgehog signaling play an important role in chondrocyte development, (hypertrophic) differentiation, and/or calcification, but their role in intervertebral disc (IVD) degeneration is unknown. Better understanding their involvement may provide therapeutic clues for low back pain due to IVD degeneration. Therefore, this study aimed to explore the role of PTHrP and hedgehog proteins in postnatal canine and human IVDs during the aging/degenerative process. The expression of PTHrP, hedgehog proteins and related receptors was studied during the natural loss of the notochordal cell (NC) phenotype during IVD maturation using tissue samples and de‐differentiation *in vitro* and degeneration by real‐time quantitative polymerase chain reaction (RT‐qPCR) and immunohistochemistry. Correlations between their expression and calcification levels (Alizarin Red S staining) were determined. In addition, the effect of PTHrP and hedgehog proteins on canine and human chondrocyte‐like cells (CLCs) was determined in vitro focusing on the propensity to induce calcification. The expression of PTHrP, its receptor (PTHR1) and hedgehog receptors decreased during loss of the NC phenotype. N‐terminal (active) hedgehog (Indian hedgehog/Sonic hedgehog) protein expression did not change during maturation or degeneration, whereas expression of PTHrP, PTHR1 and hedgehog receptors increased during IVD degeneration. Hedgehog and PTHR1 immunopositivity were increased in nucleus pulposus tissue with abundant vs no/low calcification. *In vitro*, hedgehog proteins facilitated calcification in CLCs, whereas PTHrP did not affect calcification levels. In conclusion, hedgehog and PTHrP expression is present in healthy and degenerated IVDs. Hedgehog proteins had the propensity to induce calcification in CLCs from degenerated IVDs, indicating that in the future, inhibiting hedgehog signaling could be an approach to inhibit calcification during IVD degeneration.

## Graphical abstract

Hedgehog and PTHrP expression decreases duringmaturation to increase back again in advanced stages of IVD degeneration; herein hedgehogs facilitate calcification

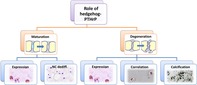

## 1 INTRODUCTION

Over 80% of the human population experiences low back pain during their lifetime.^1^ An important cause for low back pain is degeneration of the intervertebral disc (IVD).^2^ The IVD consists of an inner gelatinous nucleus pulposus (NP) and outer fibrous annulus fibrosus. During IVD maturation, large, vacuolated notochordal cells (NCs) are replaced by smaller, non‐vacuolated chondrocyte‐like cells (CLCs), a process that in the human NP has come to near completion already at birth.^3^ Thus, a “healthy” human NP from a child or young adult contains CLCs, and often no NCs, implying that the NC transition towards a CLC cellular phenotype is initially part of a maturation process. The healthy NP contains a high glycosaminoglycan (GAG) content that attracts water, whereas during IVD degeneration, the NP GAG and water content decreases and denatured collagen content increases,^4^ resulting in NP dehydration and more rigid extracellular matrix (ECM). The avascular IVD exhibits inadequate repair, leading to a vicious circle: the IVD weakens and is more vulnerable to damage by physiologic loading. Consequently, loss of mechanical function, traumatic damage, and pain develop.^5^ Current treatments for IVD disease are primarily aimed at relieving symptoms. Therefore, there is an urgent need for agents stimulating biological IVD repair.^6^ To develop such treatments, further knowledge of the pathogenesis of IVD degeneration is required.

Numerous signaling pathways have been proposed to play a role in IVD degeneration, including Wnt/β‐catenin, Sonic hedgehog (SHH), and hypoxia‐inducible factor signaling.^7^, ^8^, ^9^ SHH and Indian hedgehog (IHH) morphogens belong to the hedgehog family, which plays a crucial role in embryonic development. Hedgehog proteins undergo intramolecular cleavage catalyzed by their C‐terminal domain, yielding an N‐terminal product that represents the mature and biologically active hedgehog form and a C‐terminal product with no known signaling‐related function.^10^ SHH has been related to IVD formation and maintenance^11^ and based on this precedence, it is thought to be primarily involved in (patho)physiology of the IVD. In contrast, IHH is considered to primarily have a biologic role at the growth plate and joint cartilage level.^12^ While the regulation of hedgehog signaling is well described at the postnatal growth plate and to some extent at the joint cartilage level, the role of these two hedgehogs in postnatal IVDs of species that can suffer from clinical IVD disease remains largely elusive.

At the growth plate level, IHH and PTHrP form a growth‐restraining feedback loop regulating chondrocyte differentiation during endochondral ossification.^13^, ^14^, ^15^ IHH, produced by prehypertrophic growth plate chondrocytes, promotes proliferation and differentiation, and stimulates calcification independently from PTHrP.^16^ In the absence of hedgehogs, their receptor: Patched (PTCH1) interacts with transmembrane protein Smoothened (SMO), thereby inhibiting downstream hedgehog signaling. In the presence of hedgehog ligands, PTCH1 interacts with the ligand and is internalized. Upon this, SMO translocates to the cell membrane and activates downstream canonical hedgehog signaling, including activated target gene expression through three GLI transcription factors. GLI1 functions as transcriptional activator, whereas GLI2 and GLI3 act as either positive or negative regulators, depending on posttranscriptional and translational processing.^13^, ^14^, ^16^, ^17^, ^18^ PTHrP, produced by periarticular chondrocytes, prevents proliferative cells from leaving the proliferating growth plate zone. Moreover, targeted overexpression of PTHrP delays the appearance of hypertrophic chondrocytes.^19^ When chondrocytes are no longer sufficiently stimulated by PTHrP, they stop proliferating and synthesize IHH.^20^

Hedgehogs have been studied to some extent in joint cartilage degeneration.^21^ Considering the high homology of the biologically active N‐terminus of IHH and SHH (*ie*, 88% at the amino acid level) and the fact that these ligands share downstream signaling pathways, the reported studies are not always able to discern which of the hedgehog ligands are involved. A positive correlation between IHH or SHH expression and osteoarthritis (OA) has been described.^22^, ^23^, ^24^, ^25^ Furthermore, activated hedgehog signaling aggravated the OA phenotype in genetically modified mice.^23^ In line with this, disrupted (Indian) hedgehog signaling prevented hypertrophic chondrocyte differentiation and osteophyte formation in OA cartilage.^26^, ^27^ Furthermore, SHH may be involved in redifferentiation of transplanted chondrocytes, as de‐differentiated chondrocytes transfected with SHH showed improved cartilage repair in a rat articular cartilage defect model.^28^ Notably, PTHR1 expression was decreased in OA chondrocytes^29^ and PTHrP suppressed chondrocyte mineralization and hypertrophy,^30^ indicating that PTH(rP) signaling might prevent the development and progression of OA.^31^

**Figure 1:**
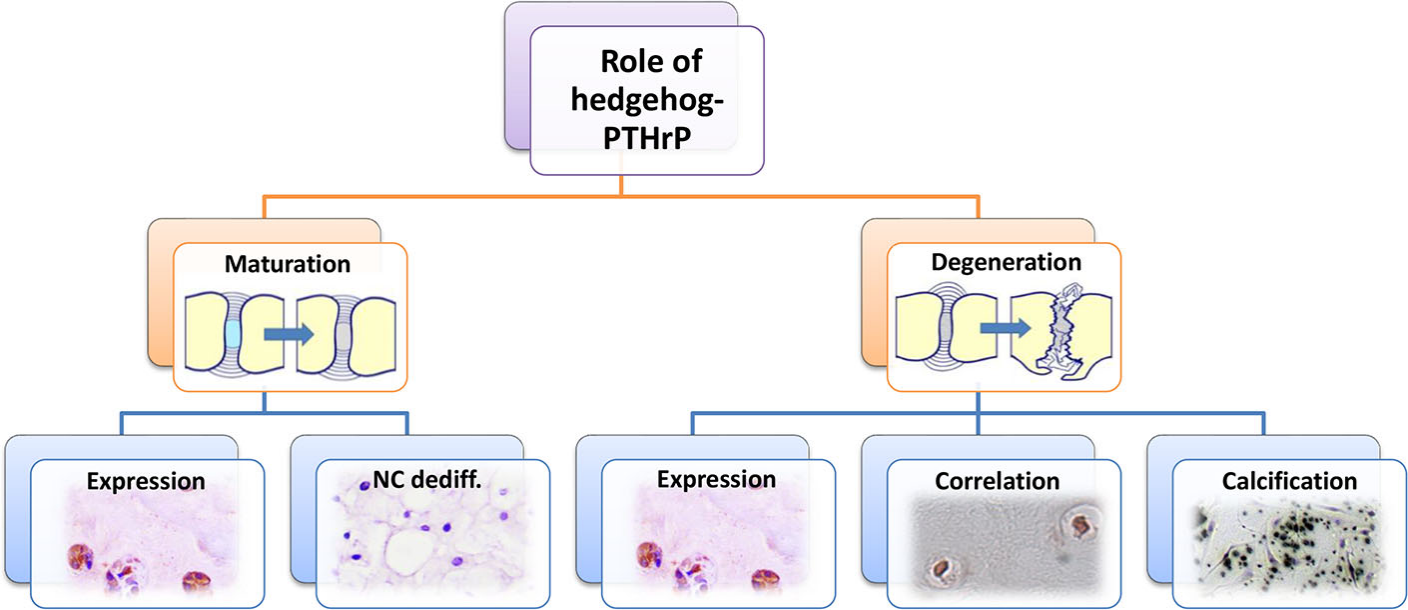
Schematic setup of the study. The role of hedgehog proteins (Indian and Sonic hedgehog; IHH and SHH, respectively) and parathyroid hormone‐related protein (PTHrP) in the intervertebral disc (IVD) was determined in two different phases: during loss of the notochordal (NC) phenotype (maturation phase) and in maturated until severely degenerated IVDs (degeneration phase). Hedgehog‐ and PTHrP‐related protein expression was determined using immunohistochemistry in canine and human NPs from healthy until severely degenerated IVDs, for example, natural IVD maturation and degeneration. To further elucidate the expression of hedgehog proteins and PTHrP during loss of the NC phenotype, IHH‐ and PTHrP‐related gene expression was studied in NCs from healthy canine IVDs that lost their specific phenotype and marker (brachyury, cytokeratin 8) expression during monolayer culture. To determine the role of hedgehog proteins and PTHrP in IVD degeneration, the correlation between PTHrP and hedgehog (receptor) expression and calcification was furthermore determined in surgically removed human NP samples across the range of histological IVD degeneration. Lastly, the effect of hedgehog proteins and PTHrP was determined on calcification of canine and human CLCs *in vitro*

Previous work indicated that IVD degeneration resembles OA, for example, hypertrophic differentiation and calcification are phenomena that can occur in both tissues during degeneration.^32^ Although hedgehog and PTHrP are extensively studied in (OA) joint cartilage, limited information is available regarding their role in the postnatal IVD. Previous work indicated that hedgehog^9^, ^33^, ^34^ and PTCH1^9^, ^35^ are expressed in the postnatal murine IVD, whereas PTHrP was absent.^9^ PTH partially recovered NC numbers in the NPs of ovariectomized rat^36^ and suppressed calcification in degenerated human CLCs.^37^ Thus, results from limited work conducted thus far imply that hedgehog proteins and PTHrP could potentially be targets for IVD repair. To take this a step further, in the present study, the expression of PTHrP and hedgehog proteins was determined in IVD maturation and degeneration by employing postnatal IVDs that can suffer from clinical IVD disease. The setup of the study is presented in Figure 1, and a schematic overview of the results is given in Table 1.

**Table 1:** Hedgehog, PTHrP, and related receptor expression during IVD maturation, degeneration and calcification

	Hedgehog	PTCH1	SMO	PTHrP	PTHR1
IVD maturation					
IHC on canine NP tissue	Not available	↓	↓	±	±
Canine NC de‐differentiation	↓ (IHH and SHH mRNA)	±	±	↓	↓
IVD degeneration					
IHC on canine NP tissue	Not available	↑^a^	↑^b^	↑	↑^b^
IHC on human NP tissue	±	↑	↑	↑	±
IVD calcification					
IHC on human NP tissue	↑	±	±	±	↑

*Note*: Hedgehog, PTHrP, and related receptor expression was studied during intervertebral disc (IVD) maturation (loss of the notochordal (NC) phenotype) using different models as given in Figure 1. Hedgehog signaling and PTHrP‐related protein expression was determined using immunohistochemistry (IHC) in nucleus pulposus (NP) tissue from healthy (Thompson score I) until maturated (Thompson score II) canine IVDs, for example, natural IVD maturation. Additionally, hedgehog‐ and PTHrP‐related mRNA expression was studied in NCs from healthy canine IVDs that de‐differentiated and lost their typical vacuolated phenotype and characteristics during monolayer culture. Furthermore, hedgehog, PTHrP and related receptor expression was studied during IVD degeneration using immunohistochemistry in NPs from maturated (Thompson score II) until severely degenerated (Thompson score V) canine and human IVDs collected during standard *postmortem* diagnostics. Lastly, the correlation between IVD calcification levels and hedgehog, PTHrP and related receptor expression was studied in surgically removed human NP tissue.

Abbreviations: CD, chondrodystrophic; NCD, non‐chondrodystrophic; PTCH1, patched, PTHrP, parathyroid hormone‐related protein, PTHR1, PTHrP receptor; SMO, smoothened; ±, not significantly affected.

a Only in NCD dogs.

b Only in CD dogs.

## 2 MATERIALS AND METHODS

### 2.1 Study design

In this study, hedgehog, PTHrP, and related receptor expression and function were determined in two different phases: during the loss of the NC phenotype (maturation phase) and in maturated until severely degenerated IVDs (degeneration phase) (Figure 1). Canine tissues and cells were employed considering the fact that they are readily available, and that dogs experience back pain and IVD degeneration with similar characteristics as humans and are a suitable translational animal model.^38^, ^39^, ^40^ Dog breeds can be classified as chondrodystrophic (CD) or non‐chondrodystrophic (NCD).^41^ CD and NCD dogs demonstrate distinct differences in clinical IVD disease which correlate with their physical appearance and are defined by their genetic background. In CD dogs, NCs are replaced by CLCs around 1 year of age and back pain due to IVD degeneration usually develops around 3 to 7 years of age. In NCD dogs, NCs can remain the predominant cell type during life and if low back pain develops, it occurs around 6 to 8 years of age.^41^

To determine the expression of possible relevant hedgehogs (*ie*, IHH and SHH) and PTHrP during loss of the NC phenotype, hedgehog‐ and PTHrP‐related gene and protein expression was studied in healthy‐maturated canine IVDs and NCs from healthy NCD canine IVDs. To determine the role of hedgehog and PTHrP in IVD degeneration, hedgehog‐ and PTHrP‐related protein expression was studied in maturated until severely degenerated canine and human IVDs. Additionally, the correlation between hedgehog, PTHrP and related receptor expression and calcification was determined in surgically removed human NP tissue across the range of histological IVD degeneration. Lastly, the effect of IHH, SHH, and PTHrP was determined on calcification of canine and human CLCs from degenerated IVDs *in vitro*.

### 2.2 Hedgehog‐PTHrP expression in cultured NC clusters

To study hedgehog and PTHrP expression during loss of the NC phenotype, hedgehog, and PTHrP related gene expression was studied in NCs from healthy NCD canine IVDs that lost their typical vacuolated phenotype and marker (brachyury, cytokeratin 8) expression during monolayer culture.^42^ This model represents NC de‐differentiation in vitro rather than the transition towards the CLC phenotype. The cervical and thoracolumbar IVDs were collected from eight NCD dogs (mixed breed, 13‐60 months) euthanized in unrelated experiments, approved by the Utrecht University Animal Ethics Committee. NC clusters were obtained as described previously^42^ and cultured in their original cluster‐like formation. Briefly, to imitate NC de‐differentiation, the NC clusters from each donor were plated in 6‐wells and cultured for 4 days at 37°C, 5% CO_2_, 21% O_2_ in DMEM‐F12 (10 565 018, Gibco), 10% fetal bovine serum (FBS, 16000‐044, Life Technologies) and 1% penicillin/streptomycin (P/S, P11‐010, PAA Laboratories). Samples for RT‐qPCR were collected at day 0, 2, and 4. RNA was isolated and cDNA was generated from the cultured NCs (*n* = 8) and native NPs (*n* = 6) as described previously.^42^ RT‐qPCR for *IHH*, *SHH*, patched *(PTCH1*), smoothened (*SMO*), parathyroid hormone‐related protein (*PTHrP*), parathyroid hormone receptor‐1 (*PTHR1*), and transcription factors *GLI1*, *GLI2*, and *GLI3* was performed and analyzed as described previously^3^ (Supporting information 1). For determination of relative quantitative gene expression, the Normfirst (*E* ^ΔΔCq^) method was used. For each target gene, the *C*_*q*_‐value of the test sample was normalized to the mean *C*_*q*_‐value of four stably expressed reference genes (*GAPDH*, *HPRT*, *RPS19*, and *SDHA*): Δ*C*_*q*_ = *C*_*q* mean ref_ − *C*_*q* target_. Secondly, the *E* ^Δ*Cq*^‐value for the test and calibrator sample was calculated. In this formula, *E* indicates the amplification efficiency of the target/reference gene and the mean *C*_*q*_‐value of all tested samples was used as calibrator. *E* ^ΔΔ*Cq*^ was calculated by normalizing the *E* ^Δ*Cq*^‐value of the test sample to one of the calibrators: *E* ^ΔΔ*Cq*^ = *E* ^Δ*Cq* test^/*E* ^Δ*Cq* calibrator^. For each target gene, the relative expression and standard deviations in gene expression were calculated.

### 2.3 Hedgehog‐PTHrP expression in healthy until severely degenerated canine and human NPs

Hedgehog‐ and PTHrP‐related immunopositivity was determined in canine and human NPs from healthy until severely degenerated (Thompson scores I‐V) IVDs. All dogs had been euthanized in unrelated experiments, approved by the Utrecht University Animal Ethics Committee, or were client‐owned dogs that were submitted for necropsy to the Faculty of Veterinary Medicine, Utrecht University. Thirty‐seven thoracolumbar or lumbosacral IVDs from dogs of various breeds (5 CD, 11 NCD), age (1‐16 years) and gender (11 female, 5 male) were studied (Supporting information 2). The samples were divided into five different grades of degeneration based on gross morphology of midsagittal sections (Thompson grading): score I is healthy and score V represents end‐stage degeneration.^43^, ^44^ IVD donors were chosen based on equal representation of all Thompson scores (*n* = 8‐7‐8‐7‐7 for grades I‐V, respectively). Tissue was obtained <24 hours after death, and IVD slices were decalcified in 0.5 M EDTA (pH 7.0) for 3 months as previously described.^45^ Sections were dehydrated and rinsed in xylene before being embedded in paraffin wax.

Human IVDs were obtained during standard postmortem diagnostics. The L2‐L5 part of the spine was collected (<48 hours after death), as approved by the scientific committee of the Pathology department (University Medical Centre Utrecht [UMCU]). Anonymous use of redundant tissue for research purposes is a standard treatment agreement with UMCU patients (Local Medical Ethical Committee number 12‐364). IVDs were used in line with the code “Proper Secondary Use of Human Tissue,” installed by the Federation of Biomedical Scientific Societies. Also, the human IVDs were chosen based on approximately equal representation of all Thompson scores (*n* = 5‐4‐4‐5‐4 for grades I‐V, respectively, Supporting information 2). IVD tissues were decalcified in Kristensen's solution (50% formic acid and 68 g/L sodium formate) in a microwave oven at 150 W and 50°C for 6 hours as previously described.^46^, ^47^ Sections were dehydrated and rinsed in xylene before embedding in paraffin wax and after hematoxylin and eosin (H&E) staining histologically graded.^48^, ^49^ Briefly, sections were scored numerically between 0 and 12 based on the presence of cell clusters, fissures, loss of demarcation and hematoxophilia (indicating reduced proteoglycan content).

For both canine and human samples, 5‐μm midsagittal sections were mounted on Microscope KP+ slides (KP‐3056, Klinipath) and immunohistochemically stained for N‐terminal hedgehog (IHH/SHH), PTCH1, SMO, PTHrP, and PTHR1 (Table 2). Negative control sections stained with blocking peptides for the specific target proteins did not show staining. End plates of each section served as internal positive control. Hedgehog proteins undergo intramolecular cleavage catalyzed by their C‐terminal domain, yielding an N‐terminal product, which represents the mature and biologically active hedgehog form and a C‐terminal product with no known signaling‐related function.^10^ The hedgehog antibody employed in the present study most probably recognizes both hedgehogs as the N‐terminus of SHH and IHH demonstrates 88% homology (pairwise alignment) up to splicing at AA 202 (IHH) or AA 198 (SHH).

**Table 2:** Details of the immunohistochemistry protocols

Target protein	Manufacturer	Concentration first Ab canine (μg/mL)	Concentration first Ab human (μg/mL)	Antibody retrieval
N‐terminal hedgehog^a^	Abcam, ab52919/ [EP1192Y]	ND	4	0.1% trypsin (30 min, 37°C).
PTCH	Santa cruz, sc‐6149	4	4	Citrate buffer (10 mM, pH 6, 30 min, 70°C)
SMO	Santa cruz, sc‐6366	4	8	Citrate buffer (10 mM, pH 6, 30 min, 70°C)
PTHrP	Santa cruz, sc‐9680	4	8	Citrate buffer (10 mM, pH 6, 30 min, 70°C)
PTHR1	Santa cruz, sc‐12 777	10	10	Citrate buffer (10 mM, pH 6, 30 min, 70°C)

*Note*: For all Santa Cruz antibodies the ImmunoCruz goat LSAB Staining System (sc‐2053, Santa Cruz) with was used. For N‐terminal hedgehog expression, EnVision+ System‐HRP Goat Anti‐Rabbit (DAKO, K4003) secondary antibody and 5% BSA/PBS (30 min) blocking was used.

Abbreviations: ND, not done, lack of reactivity to canine samples; PTCH, patched, PTHrP, parathyroid hormone‐related protein, PTHR1, PTHrP receptor; SMO, smoothened.

a Detects both N‐terminal IHH and SHH.

For all staining, raw images were captured with a Leica DFC420C digital camera (Leica Microsystems) mounted to a BX60 microscope (Olympus) and Leica Application Suite (V4.2) software package. (Positively stained) cell numbers in each NP were manually counted. Adobe Photoshop CS6 was used to manually count (positively stained) cell numbers in four (canine) or six (human) randomly selected NP areas per IVD section as described previously.^50^ The mean percentage of cells that stained positive over the total number of cells present (ratio) was determined per Thompson score for every target protein. Immunopositivity thus indicates the ratio of positive cells, not intensity of the staining. Notably, PTHrP was highly expressed only in young canine donors with healthy (Thompson score I) IVDs and its expression was considerably lower in older canine donors with healthy IVDs. To further explore the effect of age on PTHrP expression, Thompson score I IVDs of younger canine and human donors were also studied. NPs from eight canine (1 day and 6 weeks of age) and 12 human (21 weeks of gestation and 3 months of age, postnatal) donors were fixed in 4% neutral buffered formaldehyde, embedded in paraffin, and 5‐μm sections were stained for PTHrP as described previously (Table 2).

### 2.4 Correlation between PTHrP/hedgehog expression and calcification in human NPs

Surgically removed NP tissue (ethical approval was granted from Sheffield Research Ethics Committee [09/H1308/70]) was fixed in 10% v/v neutral buffered formalin and processed to paraffin wax. Following embedding, 4‐μm sections were cut and histologically graded using the previously published criteria.^48^, ^49^ To determine the frequency of calcium deposition in the NP of human IVDs, initial work investigated Alizarin Red S staining on 113 human NP samples with different grades of degeneration (Supporting information 2). Calcium deposition was then manually scored on a grade of 0 to 3, where 0 was no calcium deposits present and 3 was intense calcium (Figure 5A). Histological grading was performed by two independent blinded investigators (C.L.M. and J.S.), whereas immunohistochemistry (IHC) was analyzed by one blinded observer (C.L.M.). To enable comparison of low vs high calcium deposition, percentage of samples from non‐degenerate, mid‐grade degenerate, and high‐grade degenerate discs were then determined for those scoring ≤1 (low) and > 1 (high) calcium staining intensity. Thereafter, the correlation between PTHrP, hedgehog and related receptor expression (ratio) and calcification staining intensity was determined in a smaller subset of 30 surgically removed human NP samples with different degeneration grades and calcium deposition (Supporting information 2). N‐terminal hedgehog, PTCH1, SMO, PTHrP, and PTHR1 IHC and Alizarin Red S staining^51^ were performed as described previously.

### 2.5 The effects of hedgehog and PTHrP on calcification in CLC monolayers

Passage 2 CLCs from four canine donors (three Beagles [male], one Cocker Spaniël [female], 2‐6 years of age) and four human donors (three male, one female, 50‐63 years of age) were plated at a density of 10 000 cells/well in 12‐wells plates (665 180, Greiner) in expansion medium (hgDMEM+Glutamax [31 966, Invitrogen] with 10% FBS, 1% P/S, 0.1 mM Ascorbic acid 2‐phosphate [Asap, A8960, Sigma‐Aldrich], 10^−9^ M dexamethasone [AD1756, Sigma‐Aldrich] and 1 ng/mL bFGF [PHP105, AbD Serotec]) at 21% O_2_, 5% CO_2_, 37°C. All canine and human IVDs were mildly degenerated (Thompson grade II/III). After 1 week, expansion medium was replaced by hypertrophic induction medium (hgDMEM+Glutamax, 1% P/S, 1% ITS+ premix, 0.1 mM Asap, 10^−9^ M dexamethasone, 10 mM β‐glycerolphosphate [G9422, Sigma‐Aldrich], and 1 nM 3,3′,5‐triiodo‐L‐thyronine [T3, T6397, Sigma‐Aldrich])^52^ supplemented with/without 10^−7^ M PTHrP (PTH‐Related Protein [1‐34] amide, H‐9095, Bachem), 1 μg/mL N‐terminal IHH (1705‐HH, R&D Systems) or 1 μg/mL N‐terminal SHH (1845‐SH, R&D Systems). Concentrations for PTHrP,^53^ SHH,^54^ and IHH^55^ were chosen based on previous research. In order to facilitate hypertrophic CLC differentiation and calcification, CLCs from the same canine donors were also seeded in osteogenic culture medium (consisting of hgDMEM+Glutamax, 10% FBS, 1% P/S, 0.1 mM Asap, 10^−7^ M dexamethasone and 10 mM β‐glycerolphosphate)^51^ supplemented with/without 1 μg/mL IHH/SHH or 10^−7^ M PTHrP. After 14 days, monolayers were fixed in 4% neutral buffered formaldehyde for 2 hours. Alizarin Red S staining^51^ was performed as described previously. On the canine CLCs, also RT‐qPCR was performed as described previously^3^ for PTHR1, RUNX2, and ALP (Supporting information 1). Due to limited availability, this was not possible for human CLCs.

### 2.6 Statistical analysis

Statistical analysis was performed using IBM SPSS (version 22). All data were examined for normal distribution (Shapiro‐Wilks test). Kruskal‐Wallis and Mann‐Whitney *U* tests were performed on nonnormally distributed data, whereas general linear regression models based on ANOVAs were used for normally distributed data. To find correlations between protein immunopositivity and IVD degeneration score, partial correlations (corrected for donor) were determined. Benjamini & Hochberg False Discovery Rate post‐hoc corrections for multiple comparisons were performed. A *P*‐value <.05 was considered significant. A χ² test was performed to determine whether calcium deposition (≤1 and > 1 intensity) increased per histologic degeneration grade (≤4, 4.1‐7, and > 7).

## 3 RESULTS

### 3.1 Expression of hedgehog proteins and PTHrP during IVD maturation

To study the IVD maturation phase (defined by loss of the NC phenotype), hedgehog and PTHrP signaling‐related expression was studied in vitro in NCD canine NC‐rich NP tissue and 0 to 4 day cultured NC clusters described to de‐differentiate and lose their specific phenotype and marker (brachyury, cytokeratin 8) expression by gene expression analysis^42^ and by immunohistochemistry of the NP.

#### 3.1.1 Gene expression of canine NC cluster culture

**Figure 2:**
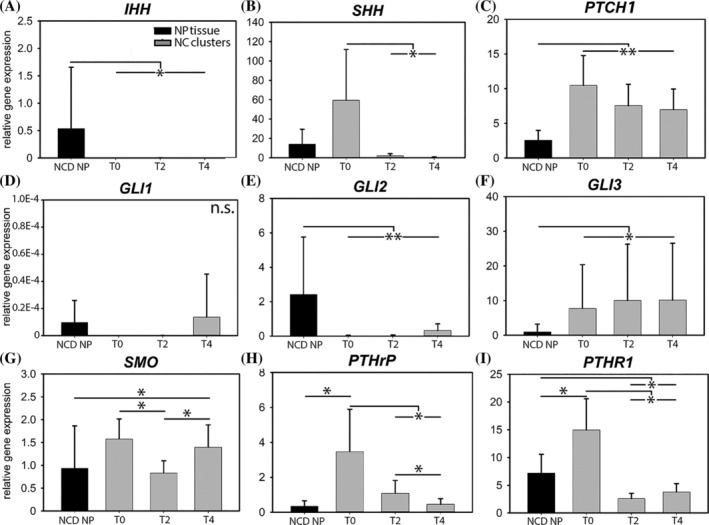
Hedgehog and PTHrP signaling‐related mRNA expression in canine nucleus pulposus (NP) tissue and cultured notochordal cell (NC) clusters. The NC clusters de‐differentiate, that is, lose their typical vacuolated phenotype and characteristics (eg, brachyury and cytokeratin 8 mRNA expression) during 4‐day monolayer culture. mRNA expression of both the hedgehog and PTHrP signaling pathway decreases with NC de‐differentiation. IHH, Indian hedgehog; NCD, non‐chondrodystrophic; n.s., not significant; PTCH1, patched, PTHrP, parathyroid hormone‐related protein, PTHR1, PTHrP receptor; SHH, Sonic hedgehog; SMO, smoothened. *n* = 6 (NP tissue) ‐ 8 (NC clusters). T0, T2, T4: NC clusters at culture day 0, 2, and 4, respectively. *P < .05 and **P < .01

*IHH* messenger RNA (mRNA) expression was significantly higher in NCD canine NP tissue than in 0‐ to 4‐day cultured NCs (*P* < 0.05), where it was hardly detectable (Figure 2A). *SHH* mRNA expression was significantly higher in non‐cultured NCs (culture day 0) and decreased rapidly during culture (*P* < .05, Figure 2B). *PTCH1* mRNA expression was significantly lower in NCD NP tissue than in 0‐ to4‐day cultured NCs (*P* < .01, Figure 2C). *SMO* mRNA expression decreased from day 0 until day 2 and thereafter increased again, and was significantly higher in 4‐day cultured NCs than in NCD NP tissue (*P* < .05, Figure 2G). mRNA of transcription factor *GLI1*, target gene of hedgehog signaling, was hardly detectable at T0 and T2, and was not differentially expressed between groups (Figure 2D). *GLI2* mRNA was expressed at significantly higher levels in NCD NP tissue than in 0‐ to 4‐day cultured NCs (*P* < .01), where it was hardly detectable (Figure 2E). mRNA expression of *GLI3* was significantly lower in NCD NP tissue than in 0‐ to ‐4 ‐day cultured NCs (*P* < .05, Figure 2F). Taken together, although *PTCH1* and *SMO* mRNA was expressed, mRNA of *IHH*, *SHH* (both hedgehog ligands) and their transcription factors *GLI1* and *GLI2* was hardly detectable (*C*_*q*_‐values > 35) in canine NCs that lost their typical vacuolated morphology during culture.

*PTHrP* mRNA expression was significantly higher in freshly isolated NCs than in native tissue (*P* < .05) and decreased rapidly during NC culture (*P* < .05, Figure 2H). Also, *PTHR1* mRNA expression decreased from day 0 to days 2 and 4 in culture (*P* < .01, Figure 2I). Furthermore, it was significantly lower in 2‐ and 4‐day cultured NCs than in NCD NP tissue (*P* < .05).

#### 3.1.2 Histology of canine and human IVD maturation and degeneration

**Figure 3:**
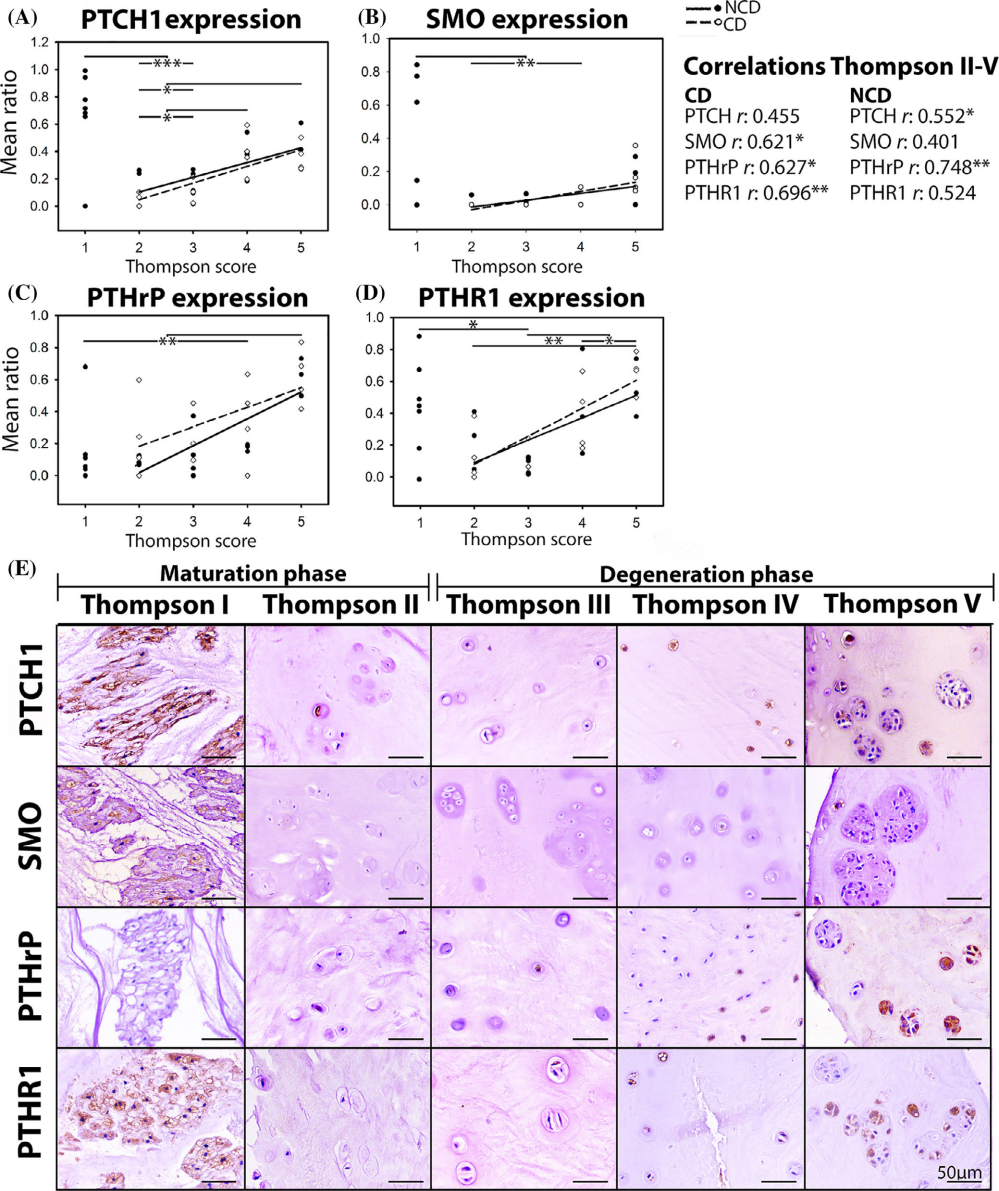
Hedgehog and PTHrP signaling‐related immunopositivity in canine intervertebral discs with different degeneration grades. Both signaling pathways are activated in healthy IVDs and increase with degeneration. The N‐terminal hedgehog antibody demonstrating active hedgehog signaling did not render specific staining in the canine samples and was therefore not included. CD, chondrodystrophic; NCD, non‐chondrodystrophic; PTCH1, patched; PTHrP, parathyroid hormone‐related protein, PTHR1, PTHrP receptor 1; SMO, smoothened. *n* = 7‐8 per Thompson score. *P < .05, **P < .01 and ***P < .001

**Figure 4:**
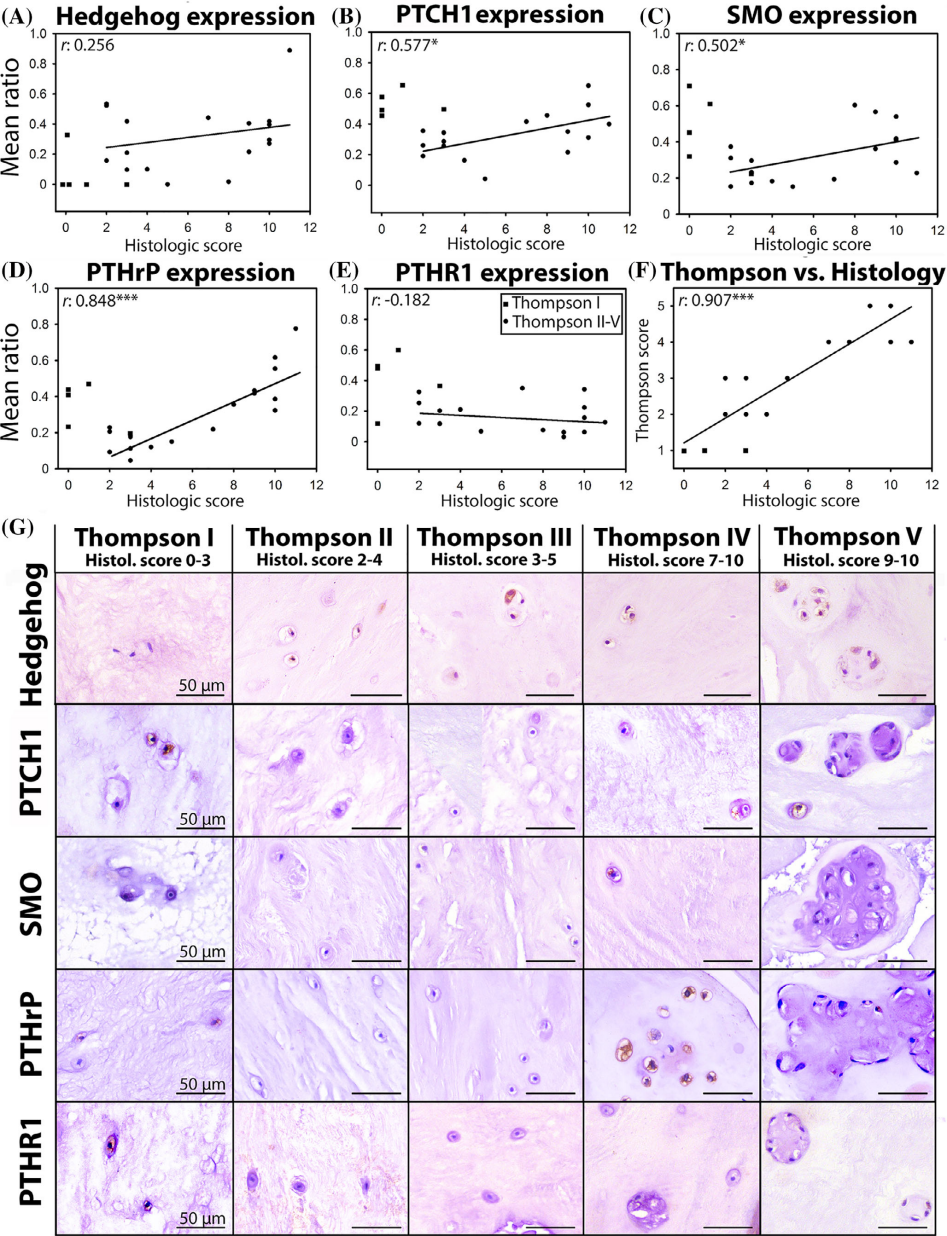
Hedgehog and PTHrP signaling‐related immunopositivity in human intervertebral discs with different degeneration grades. The samples were graded according to the macroscopic Thompson score and histologic degeneration score, which strongly correlated with each other. Both signaling pathways are increasingly activated with progress of degeneration. Hedgehog: N‐terminal active hedgehog expression, PTCH1, patched, PTHrP, parathyroid hormone‐related protein, PTHR1, PTHrP receptor 1; SMO, smoothened. *n* = 4‐5 per Thompson score. *P < .05 and ***P < .001

In healthy, Thompson score I canine NPs, only NC clusters were detected (Figure 3E). In maturated, Thompson score II canine NPs, mainly CLCs were present; only one NCD canine NP also contained some NCs. In Thompson scores III to V canine NPs, only CLCs were encountered in single cells and clusters. In NPs from healthy, Thompson score I human IVDs, mainly CLCs and only some NCs were detected, mainly as single cells, but also in clusters (Figure 4G). In NPs from Thompson scores II to V human IVDs, only CLCs were encountered in single cells and clusters. In both species, CLC clusters were more abundant and larger in NPs from Thompson score IV and V IVDs. Immunopositivity, regardless of the proteins and species, was identified in both single cells and clusters without an evident spatial distribution pattern in both species.

#### 3.1.3 Hedgehog signaling and PTHrP immunopositivity in canine IVD maturation

The N‐terminal hedgehog antibody demonstrating active hedgehog (IHH, SHH) signaling did not render specific staining in the canine samples and therefore, this antibody was only studied in human tissues. Immunopositivity (ratio of positive cells) for PTCH1 and SMO was significantly higher in canine NP tissues from healthy, Thompson score I NC‐rich IVDs than in NP tissue from maturated, Thompson score II CLC‐rich IVDs (*P* < .05, Figure 3A,B), suggesting higher expression in NCs than CLCs. PTHrP and PTHR1 immunopositivity did not differ between NPs from healthy and maturated canine IVDs (Figure 3C,D). Notably, PTHrP immunopositivity in healthy canine IVDs seemed to depend on age: PTHrP was detected in more than 10% of the NCs of 7‐ and 16‐month‐old canine donors, whereas this was 0% to 5% in older (17‐ until 96‐month‐old) canine donors (Figure 3C,E). This indicates that PTHrP was present in NCs from young, healthy canine IVDs, but that its expression decreased during aging (in contrast to the expression of PTHR1, PTCH1, and SMO). To confirm this, Thompson score I IVDs of younger canines (1 day and 6 weeks of age) were also investigated together with human (21 weeks of gestation and 3 months of age) donors as comparators. These canine NPs contained approximately 100% NCs, mainly present in clusters (Supporting information 3). In contrast, the human NPs also contained, smaller fibroblast‐ and chondrocyte‐like cells in clusters and single cells, in addition to the typical NCs (Supporting information 3). All young canine NPs showed abundant PTHrP immunopositivity in their NCs, with no clear pattern in spatial distribution. In contrast, only seven out of 12 NPs from young human donors showed PTHrP immunopositivity in both NC and CLC clusters and single cells (no spatial distribution; Supporting information 3). Taken together, this indicates that PTHrP expression is present in young, healthy NPs, but that its expression considerably decreases in older, healthy NPs, in line with results obtained using equine chondrocytes.^56^

Taken together, the mRNA expression pattern of both hedgehog and PTHrP‐signaling related genes in de‐differentiated NCs (Figure 2) were confirmed by the protein expression pattern of maturing canine IVDs (Figure 3), that is, reduced mRNA levels and immunopositivity coincided with the loss of the NC phenotype.

### 3.2 Hedgehog and PTHrP in canine and human degenerating IVDs

Regardless the species, hedgehog and PTHrP staining was cytoplasmic, while for PTCH1, SMO, and PTHR1, both the cell membrane and the cytoplasm demonstrated immunopositivity (Figures 3E and 4G). The latter seems to be counterintuitive considering the fact that PTCH1, SMO, and PTHR1 are transmembrane proteins. Nonetheless, cytoplasmic immunopositivity can be well explained for all these three proteins. Hedgehog ligands bind to their transmembrane receptor PTCH1 and cause PTCH1 inactivation, internationalization, and further degradation. Inhibition of PTCH1 is followed by SMO activation (located intracellularly) and translocation to the cell membrane, which initiates the canonical hedgehog signal transduction.^57^ As such, dependent on its localization, different forms of PTCH1 can be distinguished: (a) unliganded PTCH1, which is active and localized to the cell membrane and (b) PTCH1 bound to the ligand that is further internalized and may be further degraded.^58^ Internalization of PTCH1 is postulated to also contribute to modulation of hedgehog signaling, which remains to be further resolved. The epitope of the PTHR1 antibody maps near the N‐terminus which has been demonstrated to remain associated with its ligand within endosomes, challenging the classical paradigm of G protein‐coupled receptor signaling.^59^ Considering the semi‐quantitative nature of immunostainings and technical challenges concerning quantification, follow‐up analysis of immunostainings did not distinguish between the different patterns of immunopositivity.

#### 3.2.1 Degenerating canine IVDs

Considering the differential clinical representation of IVD disease among dog breeds,^41^ the canine IVDs were separately analyzed for CD and NCD dogs. PTCH1 immunopositivity, a direct target of active hedgehog signaling, was significantly higher in NPs from Thompson scores IV to V canine IVDs than in NPs from Thompson score II to III IVDs (*P* < .05, Figure 3A). Only NCD dogs showed a positive correlation between PTCH1 immunopositivity and macroscopic IVD degeneration grade (Thompson scores II‐V, *P* < .05, *r*:0.552), whereas only CD dogs showed a positive correlation between SMO immunopositivity and macroscopic IVD degeneration grade (*P* < .05, *r*:0.621). PTHrP immunopositivity significantly increased during canine IVD degeneration (*P* < .01, Figure 3C). In both CD and NCD dogs, a positive correlation between PTHrP immunopositivity and macroscopic IVD degeneration grade was observed (*P* < .05, *r*:0.627 [CD] and *P* < .01, *r*:0.748 [NCD]). Immunopositivity for PTHR1 increased from NPs of maturated, Thompson scores II to III IVDs to severely degenerated, Thompson score V IVDs (*P* < .05, Figure 3D). Only CD dogs showed a positive correlation between PTHR1 immunopositivity and macroscopic IVD degeneration grade (*P* < .01, *r*:0.696). Although the current study demonstrates some differences in hedgehog‐PTHrP (related) expression between CD and NCD dogs, which may be related with their differential genetic background, the direction of the correlation between protein expression and IVD degeneration grade was similar.

#### 3.2.2 Human degenerating IVDs

As expected, a strong, positive correlation was found between the macroscopic Thompson and the histologic IVD degeneration score^48^, ^49^ of the human samples collected *postmortem* (*r*:0.907, *P* < .001; Figure 4F). To be able to relate these results to the follow‐up analysis (*ie*, the relationship between PTHrP/hedgehog immunopositivity and calcification in surgically removed human NP samples), correlation are given in relation to the histological IVD degeneration grade. A significant positive correlation between PTCH1, SMO, and PTHrP immunopositivity and histologic IVD degeneration grade was encountered (*P* < .05, *r*:0.679, 0.577, 0.502, and 0.848, respectively; Figure 4B‐D). There was, however, no significant correlation between N‐terminal hedgehog or PTHR1 immunopositivity and histologic IVD degeneration grade (Figure 4A, E).

### 3.3 Hedgehog and PTHrP in IVD calcification

To further explore the role of hedgehog and PTHrP in more clinically relevant material, immunopositivity was correlated to calcification in surgically removed human NP samples across the range of histological IVD degeneration.^48^, ^49^ Lastly, the effect of IHH and SHH was determined on calcification of canine and human CLCs *in vitro*. Initial experiments in canine CLCs (*n* = 4 Beagles) demonstrated that PTHrP did not induce calcifications under the conditions studied (Supporting information 5) and was further disregarded for follow‐up culture experiments.

**Figure 5:**
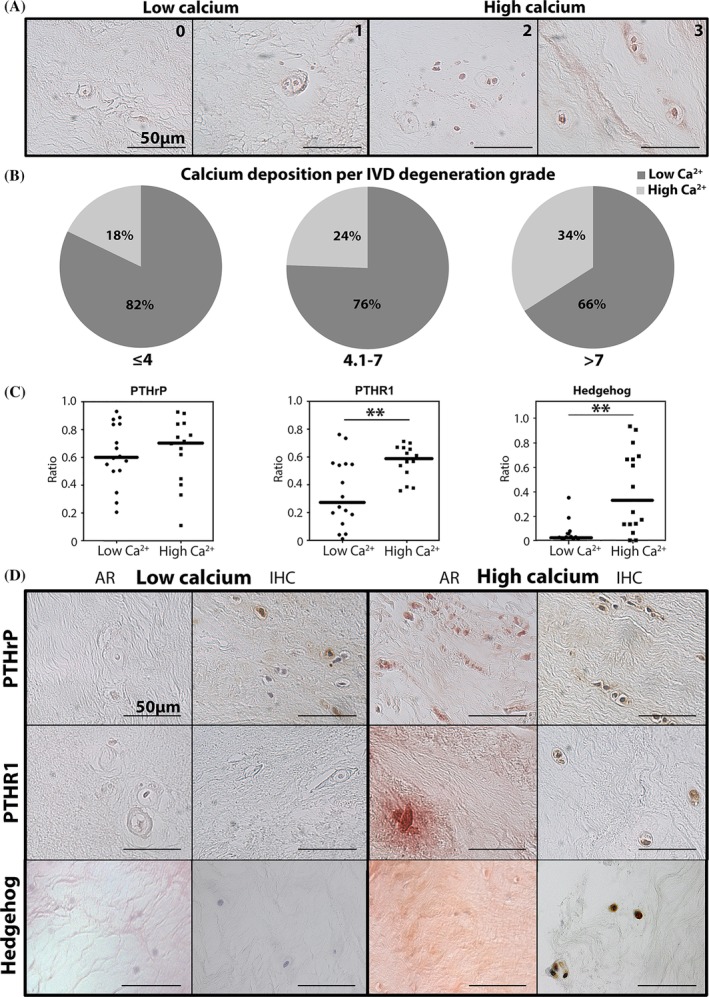
PTHrP, PTHR1, and N‐terminal hedgehog immunopositivity and calcium deposition in surgically removed human nucleus pulposus tissue across the range of histological IVD degeneration. The degree of histologic IVD degeneration was divided into three groups (≤4, 4.1‐7, and > 7), while calcium deposition was divided into low (≤1) and high (≥1) intensity. (A) Alizarin Red S staining of representative samples with different calcium staining intensity (0‐3). (B) Calcium deposition increases with the degree of histologic IVD degeneration. *n* = 133 (C) PTHrP, PTHR1, and N‐terminal hedgehog immunopositivity (ratio) vs calcium deposition (low (≤1) and high (≥1) intensity). Bars indicate median. *n* = 30 (D) Alizarin Red S staining (AR; left) and immunohistochemistry (IHC; right) for PTHrP, PTHR1, and N‐terminal hedgehog of representative human NP samples with low (≤1) and high (≥1) calcium deposition. Hedgehog: N‐terminal active hedgehog expression, PTHrP, parathyroid hormone‐related protein, PTHR1, PTHrP receptor 1, **P < .01

The morphological assessment of the calcium staining intensity was divided into two groups (≤1 and > 1 staining intensity; Figure 5A). The percentage of human NP tissue samples with high calcium deposition significantly increased with the histologic IVD degeneration grade (Figure 5B; *P* < .05). Moreover, the number of cells with PTHR1 and N‐terminal hedgehog immunopositivity was significantly higher in the NP tissues associated with high calcium deposition (*P* < .01; Figure 5C,D). In contrast, no difference in number of immunopositive cells for PTHrP (Figure 5C,D), PTCH1 and SMO (data not shown) was observed between the NPs with low or high calcium deposition.

**Figure 6:**
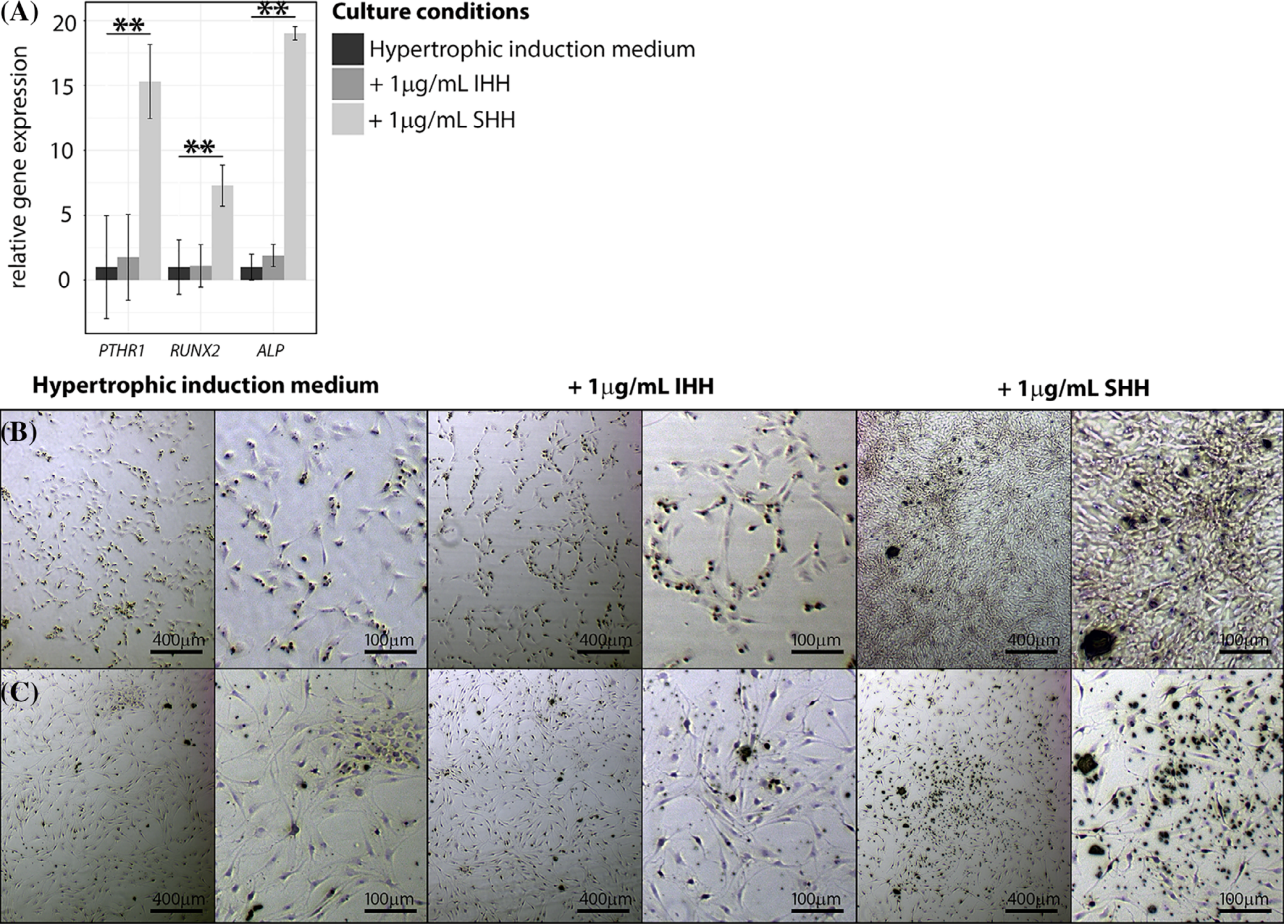
Indian and Sonic hedgehog facilitate calcification in human and canine chondrocyte‐like cells in vitro. (A) Hedgehog and PTHrP signaling‐related mRNA expression in canine CLC monolayers treated with hypertrophic induction medium supplemented with/without 1 μg/mL IHH or SHH for 7 days. *n* = 4. **P < .01. ALP, alkaline phosphatase; IHH, Indian hedgehog; PTHR1, PTHrP receptor 1, RUNX2, Runt‐related transcription factor 2; SHH, Sonic hedgehog. Alizarin Red S staining on one representative canine (B) and human (C) CLC donor. CLC monolayers were treated with hypertrophic induction medium supplemented with/without 1 μg/mL IHH or SHH for 7 days. *n* = 4 per species

Short‐term CLC monolayer culture has previously been employed as a model to study calcium deposition.^37^, ^60^ Interestingly, the canine CLCs seeded in osteogenic culture medium did not thrive; they failed to attach to culture plastic and died within 24 hours. Canine CLCs treated with hypertrophic induction medium + SHH demonstrated increased gene expression of (pre)hypertrophic differentiation markers *PTHR1*, *RUNX2*, and *ALP* compared with hypertrophic induction medium alone and hypertrophic induction medium + IHH (Figure 6A). Affirmatively, hypertrophic induction medium alone induced calcification in canine and human CLC monolayers, while the supplementation of SHH exerted a more pronounced effect in this respect compared to IHH (Figure 6B,C and Supporting information 4).

## 4 DISCUSSION

Treatment strategies leading to functional IVD restoration can benefit from in‐depth knowledge of the pathways involved in IVD degeneration. Therefore, this study focused on the largely unexplored role of PTHrP and hedgehog proteins in the postnatal IVD of species that can suffer from clinical IVD disease. The current manuscript provides the first step towards a better understanding of the possible involvement of PTHrP and hedgehog proteins in IVD maturation, degeneration, and calcification.

### 4.1 Hedgehog and PTHrP expression decreases during IVD maturation

To study hedgehog‐ and PTHrP‐related mRNA and protein expression during loss of the NC phenotype, NC‐rich healthy and CLC‐rich maturated canine IVDs as well as canine NCs that de‐differentiated during monolayer culture^7^ were studied. The results from those two models indicate that the expression of SHH/IHH, PTHrP and their receptors was high in healthy, NC‐containing NPs, but decreased with the transition towards maturated, CLC‐containing NPs. Whether the observed changes in expression are a cause or consequence of the maturation process; play a role in preservation of NCs; or are related to NP differentiation, remains to be determined with the aid of antagonists or functional knock‐out/in models.

### 4.2 Hedgehog and PTHrP expression increases from maturated towards severely degenerated IVDs

PTCH1, SMO (both direct targets of active hedgehog signaling) and PTHrP expression increased during the IVD degeneration process in the studied species. This result is in line with the positive correlation between IHH expression and OA,^22^, ^23^, ^24^, ^25^ a process resembling IVD degeneration.^32^ PTHR1 immunopositivity increased from maturated to severely degenerated canine, but not human IVDs. While a species‐specific difference cannot be excluded, the different decalcification procedures of the IVD segments may have affected the results.^61^ This plausible explanation is further underscored by the observation that non‐decalcified human NP samples collected during surgery demonstrated increased PTHR1 immunopositivity in direct relation to calcium deposition levels seen in higher frequencies in degenerated NPs. Altogether, the descriptive results from the present study indicate that activated hedgehog and PTHrP signaling are involved in IVD degeneration. Therefore, functional in vitro studies were performed to explore the role of PTHrP and hedgehogs in degenerated IVDs.

### 4.3 Calcium levels increase in the human NP during IVD degeneration

Based on their well‐described role in chondrocytes,^22^, ^23^, ^24^, ^30^ hedgehog proteins and PTHrP could be involved in hypertrophic CLC differentiation and calcification, processes that are known to occur during the later stages of IVD degeneration.^32^, ^62^, ^63^, ^64^ The present study confirmed a positive correlation between human NP calcification levels and IVD degeneration grade. A significant, positive correlation between N‐terminal hedgehog immunopositivity and calcification was detected in human NP tissue collected during therapeutic surgical procedures. In line with this, previous work displayed a significant association between IHH and calcification in OA samples.^22^, ^23^, ^24^ PTHR1 immunopositivity was also significantly higher in NPs with abundant calcification vs those with no/low calcification, which can be explained by the cellular phenotype: PTHR1 is mainly expressed in prehypertrophic cells^65^ and calcium is most likely deposited in severely degenerated IVDs^32^, ^62^, ^63^ with (pre)hypertrophically differentiated CLCs. Previous work showed that PTH inhibits terminal differentiation of chondrocytes^31^ and calcification in CLCs^37^ and that PTHrP inhibits calcification in chondrocytes.^30^ In contrast, in the present study, PTHrP immunopositivity was similar between NPs with low or abundant calcification and PTHrP did not affect calcification of CLCs in vitro. However, assuming that PTHrP inhibits the IVD calcification process in vivo, the absence of PTHrP upregulation in later stages of degeneration even in the presence of high PTHR1 levels may allow for NP calcification in the process of degeneration.

### 4.4 SHH and IHH induce calcification in canine and human CLCs

In contrast to PTHrP, IHH is known to promote chondrocyte hypertrophy and calcification in OA.^22^, ^23^, ^24^ To the best of our knowledge, this is the first study to demonstrate that IHH and especially SHH also have the propensity to induce calcification in human and canine CLCs from degenerated IVDs *in vitro*. Interestingly, SHH was even more potent in inducing calcification in human and canine CLCs than IHH. Together with the increased hedgehog expression in IVDs with high levels of calcium, this may suggest a possible role for SHH and/or IHH in calcification during IVD degeneration. In line with this thought, disrupted IHH signaling prevented hypertrophic chondrocyte differentiation and osteophyte formation in OA cartilage.^26^, ^27^ Possibly, this could also be a therapeutic approach to prevent or retard these processes during IVD degeneration. From a clinical perspective, however, additional considerations need to be acknowledged, including possible side effects of inhibited hedgehog signaling, for example, on the cardiovascular^66^ and central nervous^67^ system.

## 5 CONCLUSIONS

Expression of hedgehogs, PTHrP and their receptors were high in NCs from young, healthy IVDs, decreased together with the loss of the NC phenotype, and increased again in the advanced stages of IVD degeneration. While PTHrP did not affect calcification, IHH and mainly SHH had the propensity to facilitate calcification in human and canine CLCs *in vitro*. Taken together with the increased N‐terminal hedgehog immunopositivity in IVDs with high levels of calcium deposition, this may indicate that inhibition of hedgehog signaling could be a therapeutic approach to inhibit CLC calcification in IVD degeneration.

## CONFLICT OF INTEREST

The authors declare no potential conflict of interest.

## AUTHOR CONTRIBUTIONS

The manuscript has been read and approved by all authors. There are no other persons who satisfied the criteria for authorship. Each author has contributed to a minimum of two of the four major parts of the submitted work and there are no “Gift Authorships.” Given the extent of the study, nine authors have been listed. They contributed as follows: F.C.B. collected material, performed most of the experiments, mined, and analyzed the data, participated in the design of the study, and drafted the manuscript. K.M.R., F.M.R., J.W.S., C.L.M., W.A.M.J., and Y.Z. assisted in performance of experiments and mining the data. L.B.C. and C.L.M. provided human IVDs. L.B.C., C.L.M., and D.C. contributed to the study design and data analysis. M.A.T. conceived the study, mined and analyzed data, assisted in drafting the manuscript and coordinated the process. All authors read and approved the final manuscript.

## Supporting information


**Supporting information 1** Primers used for quantitative PCR of canine samples

**Supporting information 2** Canine and human IVD donors for immunohistochemistry

**Supporting information 3** PTHrP immunopositivity in young canine and human NP tissue

**Supporting information 4** Indian and Sonic hedgehog facilitate calcification in human and canine chondrocyte‐like cells in vitro.

**Supporting information 5** PTHrP does not facilitate calcification in canine chondrocyte‐like cells in vitro.
