# Supplementary material for: Hedgehog proteins and parathyroid hormone‐related protein are involved in intervertebral disc maturation, degeneration, and calcification
Source: JOR Spine. 2019 Nov 19;2(4):e1071. doi: 10.1002/jsp2.1071 (PMC6920702; doi:10.1002/jsp2.1071)
Supplement: Supplementary file 1 — Supporting information 1 Primers used for quantitative PCR of canine samples [file JSP2-2-e1071-s001.docx]

**Supporting information 1. Primers used for quantitative PCR of canine samples**

| **Genes** | **Forward sequence 5’ → 3’** | **Reverse sequence 5’ → 3’** | **Amplicon size** |
| --- | --- | --- | --- |
| **Reference genes** |  |  |  |
| *GAPDH* | TGTCCCCACCCCCAATGTATC | CTCCGATGCCTGCTTCACTACCTT | 100 |
| *HPRT* | AGCTTGCTGGTGAAAAGGAC | TTATAGTCAAGGGCATATCC | 104 |
| *RPS19* | CCTTCCTCAAAAAGTCTGGG | GTTCTCATCGTAGGGAGCAAG | 95 |
| *SDHA* | GCCTTGGATCTCTTGATGGA | TTCTTGGCTCTTATGCGATG | 92 |
| **Target genes** |  |  |  |
| *ACAN* | GGACACTCCTTGCAATTTGAG | GTCATTCCACTCTCCCTTCTC | 111 |
| *ALP* | GGCTTCAGAATCTCAACAC | AACTTGTCCATCTCCAGC | 150 |
| *COL2A1* | GCAGCAAGAGCAAGGAC | TTCTGAGAGCCCTCGGT | 151 |
| *GLI1* | TCAAGGCTCAGTACATGCTG | ATGGCTTCTCATTGGAGTGG | 240 |
| *GLI2* | CACGCTCTGGGAAATGAGG | CGGGCATCAGCAACATG | 145 |
| *GLI3* | CCAGCAGGAACAGCCAG | GAACTCCTTCTTCTCGCCG | 190 |
| *IHH** | TCACCACTCAGAGGAGTCG | GTGCTCAGACTTGACGGAG | 172 |
| *PTCH1* | CCTCCTCATATTTGGGGC | CACCTTCTTCTTTCGGGG | 158 |
| *PTHrP* | GTGTTCCTGCTGAGCTACTCG | ATGGGTGGTCGCCTTCTA | 451 |
| *PTHR1* | GACCACATCCTTTGCTGG | CAAACACCTCCCGTTCAC | 217 |
| *RUNX2* | AACGATCTGAGATTTGTGGGC | TGTGATAGGTGGCTACTTGGG | 97 |
| *SHH** | CAGTGGAAGATACGAAGGGA | TTGTCCTTGCACCTCTGAG | 140 |
| *SMO* | CTATGTGCTGTGCCAG | ATCACTCTGCCCAGTC | 214 |

* Primers for hedgehogs are specific and do not amplify other hedgehog variants.
