# Supplementary material for: Hedgehog proteins and parathyroid hormone‐related protein are involved in intervertebral disc maturation, degeneration, and calcification
Source: JOR Spine. 2019 Nov 19;2(4):e1071. doi: 10.1002/jsp2.1071 (PMC6920702; doi:10.1002/jsp2.1071)
Supplement: Supplementary file 2 — Supporting information 2 Canine and human IVD donors for immunohistochemistry [file JSP2-2-e1071-s002.docx]

**Supporting information 2. Canine and human IVD donors for immunohistochemistry**

**Canine IVD donors for hedgehog-PTHrP-related IHC – Figure 3**

| Dog number | IVD level | Age (months) | CD/NCD | Breed |
| --- | --- | --- | --- | --- |
| Thompson score I | | | | |
| 1 | T12/T13 | 16 | NCD | Flatcoated retriever |
| 2 | T11/T12 | 7 | NCD | Mongrel |
| 3 | T12/T13 | 36 | NCD | Kerry beagle |
| 4 | L7/S1 | 36 | NCD | Kerry beagle |
| 5 | L5/L6 | 96 | NCD | Kerry beagle |
| 3 | L4/L5 | 36 | NCD | Kerry beagle |
| 2 | T13/L1 | 16 | NCD | Mongrel |
| 6 | L4/L5 | 17 | NCD | Mongrel |
| Thompson score II | | | | |
| 7 | L7/S1 | 25 | CD | Beagle |
| 2 | L4/L5 | 16 | NCD | Mongrel |
| 8 | L6/L7 | 28 | CD | Beagle |
| 9 | L4/L5 | 84 | NCD | Foxhound |
| 10 | L3/L4 | 25 | CD | Beagle |
| 9 | L5/L6 | 84 | NCD | Foxhound |
| 7 | L3/L4 | 25 | CD | Beagle |
| Thompson score III | | | | |
| 11 | L7/S1 | 117 | CD | Beagle |
| 11 | L6/L7 | 117 | CD | Beagle |
| 9 | T13/L1 | 84 | NCD | Foxhound |
| 12 | T12/T13 | 120 | NCD | Foxhound |
| 12 | L2/L3 | 120 | NCD | Foxhound |
| 7 | T13/L1 | 25 | CD | Beagle |
| 12 | T13/L1 | 120 | NCD | Foxhound |
| 13 | L7/S1 | 142 | NCD | Bouvier |
| Thompson score IV | | | | |
| 9 | L7/S1 | 84 | NCD | Foxhound |
| 14 | L2/L3 | 120 | CD | Beagle |
| 15 | L7/S1 | 108 | NCD | Foxhound |
| 14 | T13/L1 | 120 | CD | Beagle |
| 12 | T11/T12 | 120 | NCD | Foxhound |
| 14 | L7/S1 | 120 | CD | Beagle |
| 14 | L1/L2 | 120 | CD | Beagle |
| Thompson score V | | | | |
| 14 | L5/L6 | 120 | CD | Beagle |
| 14 | T11/T12 | 120 | CD | Beagle |
| 14 | T12/T13 | 120 | CD | Beagle |
| 16 | L7/S1 | 192 | CD | Welsh Terriër |
| 16 | L1/L2 | 192 | CD | Welsh Terriër |
| 14 | L4/L5 | 120 | CD | Beagle |
| 16 | T11/T12 | 192 | CD | Welsh Terriër |

**Human IVD donors for hedgehog/PTHrP-related IHC (level L3-L4) – Figure 4**

| Number | Age (years) | Gender | Histologic score |  |  |
| --- | --- | --- | --- | --- | --- |
| Thompson score I | | |  |  |  |
| 1 | 17 | Male | 0 |  |  |
| 2 | 21 | Female | 0 |  |  |
| 3 | 14 | Female | 1 |  |  |
| 4 | 14 | Male | 0 |  |  |
| 5 | 18 | Female | 3 |  |  |
| Thompson score II | | |  |  |  |
| 6 | 63 | Male | 4 |  |  |
| 7 | 35 | Male | 3 |  |  |
| 8 | 50 | Female | 2 |  |  |
| 9 | 39 | Male | 2 |  |  |
| Thompson score III | | |  |  |  |
| 10 | 73 | Female | 5 |  |  |
| 11 | 80 | Female | 3 |  |  |
| 12 | 46 | Female | 3 |  |  |
| 13 | 59 | Female | 4 |  |  |
| Thompson score IV | | |  |  |  |
| 14 | 88 | Female | 10 |  |  |
| 15 | 73 | Female | 7 |  |  |
| 16 | 84 | Male | 11 |  |  |
| 17 | 70 | Female | 8 |  |  |
| 18 | 77 | Male | 10 |  |  |
| Thompson score V | | |  |  |  |
| 19 | 62 | Female | 10 |  |  |
| 20 | 59 | Male | 10 |  |  |
| 21 | 71 | Male | 9 |  |  |
| 22 | 88 | Female | 9 |  |  |

**Human NP tissue donors for Alizarin Red S and hedgehog/PTHrP-related IHC – Figure 5**

| Number | Source | Age | IVD Level | Histological Grade | Classification | Alizarin Red S | IHC |
| --- | --- | --- | --- | --- | --- | --- | --- |
| 1 | Surgical | 42 | L4/L5 | 3 | Non-degenerate | ✓ | ✓ |
| 2 | Surgical | 40 | L5/S1 | 3.9 | Non-degenerate | ✓ |  |
| 3 | Surgical | 25 | L4/L5 | 4.8 | Degenerate | ✓ |  |
| 4 | Surgical | 50 | L4/L5 | 4 | Non-degenerate | ✓ |  |
| 5 | Surgical | 33 | L5/S1 | 9 | Degenerate | ✓ |  |
| 7 | Surgical | 47 | C6/C7 | 5 | Degenerate | ✓ |  |
| 8 | Surgical | 70 | L4/L5 | 7.5 | Degenerate | ✓ |  |
| 9 | Surgical | 32 | L5/S1 | 5 | Degenerate | ✓ |  |
| 15 | Surgical | 34 | L4/L5 | 7.5 | Degenerate | ✓ |  |
| 19 | Surgical | 36 | L5/S1 | 9 | Degenerate | ✓ |  |
| 24 | Surgical | 35 | L4/L5 | 2 | Non-degenerate | ✓ | ✓ |
| 27 | Surgical | 73 | L4/L5 | 4.7 | Degenerate | ✓ |  |
| 30 | Post mortem | 45 | L5/S1 | 2 | Non-degenerate | ✓ | ✓ |
| 31 | Post mortem | 45 | L3/L4 | 1 | Non-degenerate | ✓ | ✓ |
| 32 | Post mortem | 45 | L3/L4 | 4 | Non-degenerate | ✓ | ✓ |
| 33 | Surgical | 48 | L4/L5 | 8 | Degenerate | ✓ |  |
| 36 | Surgical | 33 | L5/S1 | 9 | Degenerate | ✓ |  |
| 37 | Post mortem | 74 | L5/S1 | 3 | Non-degenerate | ✓ |  |
| 38 | Post mortem | 74 | L4/L5 | 5 | Degenerate | ✓ | ✓ |
| 39 | Post mortem | 74 | L3/L4 | 11.5 | Degenerate | ✓ | ✓ |
| 40 | Post mortem | 74 | L2/L3 | 11 | Degenerate | ✓ | ✓ |
| 44 | Surgical | 42 | L5/S1 | 2 | Non-degenerate | ✓ | ✓ |
| 45 | Surgical | 36 | L5/S1 | 8 | Degenerate | ✓ |  |
| 46 | Surgical | 41 | L5/S1 | 8.5 | Degenerate | ✓ |  |
| 52 | Surgical |  | L4/L5 | 9 | Degenerate | ✓ |  |
| 53 | Surgical | 38 | L5/S1 | 7 | Degenerate | ✓ |  |
| 55 | Surgical |  | L5/S1 | 6 | Degenerate | ✓ |  |
| 56 | Surgical | 43 | L5/S1 | 8 | Degenerate | ✓ |  |
| 57 | Surgical | 44 | L5/S1 | 9 | Degenerate | ✓ |  |
| 60 | Surgical | 38 | L5/S1 | 6 | Degenerate | ✓ |  |
| 63 | Surgical | 42 | L5/S1 | 5 | Degenerate | ✓ |  |
| 65 | Surgical | 43 | L4/L5 | 10 | Degenerate | ✓ | ✓ |
| 66 | Surgical | 62 | L3/L4 | 10 | Degenerate | ✓ |  |
| 67 | Surgical | 39 | L4/L5 | 5 | Degenerate | ✓ |  |
| 68 | Surgical | 62 | L4/L5 | 9 | Degenerate | ✓ |  |
| 69 | Surgical | 37 | L5/S1 | 8.5 | Degenerate | ✓ |  |
| 70 | Surgical | 39 | L5/S1 | 7.5 | Degenerate | ✓ |  |
| 71 | Surgical | 42 | L5/S1 | 3 | Non-degenerate | ✓ |  |
| 72 | Surgical |  | C5/C6 | 9.5 | Degenerate | ✓ |  |
| 74 | Surgical |  | L4/L5 | 7.5 | Degenerate | ✓ |  |
| 75 | Surgical | 40 | L3/L4 | 11 | Degenerate | ✓ | ✓ |
| 78 | Surgical | 54 | C3/C4 | 10 | Degenerate | ✓ |  |
| 80 | Surgical | 33 | L5/S1 | 5 | Degenerate | ✓ | ✓ |
| 84 | Surgical | 38 | C6/C7 | 5 | Degenerate | ✓ |  |
| 85 | Surgical | 85 | L2/L3 | 8 | Degenerate | ✓ |  |
| 86 | Surgical | 40 | L5/S1 | 9 | Degenerate | ✓ |  |
| 89 | Surgical | 21 | L5/S1 | 4 | Non-degenerate | ✓ | ✓ |
| 90 |  |  |  | 11 | Degenerate | ✓ | ✓ |
| 93 | Surgical | 38 | L5/S1 | 12 | Degenerate | ✓ | ✓ |
| 95 | Surgical | 38 | L5/S1 | 6 | Degenerate | ✓ |  |
| 97 | Surgical | 46 | L5/S1 | 10 | Degenerate | ✓ |  |
| 100 | Surgical | 43 | L5/S1 | 7 | Degenerate | ✓ |  |
| 101 | Surgical | 54 | C5/C6 | 4.5 | Degenerate | ✓ |  |
| 103 | Surgical | 45 | C5/C6 | 9.5 | Degenerate | ✓ |  |
| 104 | Surgical | 33 | L5/S1 | 5.5 | Degenerate | ✓ |  |
| 109 | Surgical | 29 | L4/L5 | 7 | Degenerate | ✓ |  |
| 110 | Surgical | 24 | L3/L4 | 3 | Non-degenerate | ✓ |  |
| 111 | Surgical | 68 | L4/L5 | 8 | Degenerate | ✓ |  |
| 112 | Surgical | 42 | L5/S1 | 11 | Degenerate | ✓ | ✓ |
| 118 | Surgical | 27 | L4/L5 | 8 | Degenerate | ✓ |  |
| 121 | Surgical | 27 | L4/L5 | 3 | Non-degenerate | ✓ |  |
| 122 | Surgical | 29 | L4/L5 |  |  | ✓ |  |
| 124 | Surgical | 40 | L4/L5 | 3.5 | Non-degenerate | ✓ | ✓ |
| 127 | Surgical | 27 | L5/S1 | 4 | Non-degenerate | ✓ |  |
| 133 | Surgical | 35 | L4/L5 | 7 | Degenerate | ✓ |  |
| 134 | Surgical | 54 | C5/C6 | 9 | Degenerate | ✓ |  |
| 136 | Surgical | 33 | L4/L5 |  |  | ✓ |  |
| 144 | Surgical | 37 | L5/S1 | 9 | Degenerate | ✓ |  |
| 145 | Surgical | 38 | L4/L5 | 11 | Degenerate | ✓ | ✓ |
| 146 | Surgical | 47 | L5/S1 | 7 | Degenerate | ✓ |  |
| 154 | Surgical | 52 | L4/L5 | 11 | Degenerate | ✓ |  |
| 157 | Surgical | 31 | L5/S1 | 5 | Degenerate | ✓ |  |
| 159 | Surgical | 39 | L4/L5 | 9 | Degenerate | ✓ |  |
| 161 | Surgical | 39 | C6/C7 | 7 | Degenerate | ✓ |  |
| 170 | Surgical |  | L5/S1 | 10 | Degenerate | ✓ | ✓ |
| 174 | Surgical | 29 | L5/S1 | 6 | Degenerate | ✓ |  |
| 175 | Surgical | 37 | L5/S1 | 7 | Degenerate | ✓ |  |
| 177 | Surgical | 51 | L4/L5 | 2 | Non-degenerate | ✓ | ✓ |
| 179 | Surgical | 79 | C4/C5 | 4 | Non-degenerate | ✓ | ✓ |
| 184 | Surgical | 49 | L4/L5 | 5 | Degenerate | ✓ |  |
| 192 | Surgical | 29 | L5/S1 | 8 | Degenerate | ✓ |  |
| 194 | Surgical | 35 | L4/L5 | 11 | Degenerate | ✓ |  |
| 197 | Surgical | 42 | L4/L5 | 8 | Degenerate | ✓ |  |
| 203 | Surgical | 45 | L5/S1 | 4 | Non-degenerate | ✓ |  |
| 207 | Surgical | 38 | L5/S1 | 8 | Degenerate | ✓ |  |
| 225 | Surgical | 36 | L5/S1 | 10 | Degenerate | ✓ | ✓ |
| 228 | Surgical |  |  | 7 | Degenerate | ✓ |  |
| 229 | Surgical | 56 | L4/L5 | 6 | Degenerate | ✓ |  |
| 230 | Surgical | 56 | L5/S1 | 8.5 | Degenerate | ✓ |  |
| 231 | Surgical | 56 | L4/L5 | 5 | Degenerate | ✓ |  |
| 232 | Surgical | 58 | L5/S1 | 8 | Degenerate | ✓ |  |
| 233 | Surgical | 44 | L5/S1 | 10 | Degenerate | ✓ | ✓ |
| 234 | Surgical | 54 | L5/S1 | 9 | Degenerate | ✓ | ✓ |
| 243 | Surgical | 28 | L4/L5 | 6 | Degenerate | ✓ |  |
| 244 | Surgical | 43 | L4/L5 | 4 | Non-degenerate | ✓ |  |
| 245 | Surgical | 80 | L4/L5 | 4 | Non-degenerate | ✓ | ✓ |
| 253 | Surgical | 63 | C5/C6 | 4 | Non-degenerate | ✓ |  |
| 254 | Surgical | 47 | L4/L5 | 6 | Degenerate | ✓ |  |
| 257 | Surgical | 46 | L5/S1 | 3 | Non-degenerate | ✓ | ✓ |
| 263 |  |  |  | 9 | Degenerate | ✓ | ✓ |
| 264 | Surgical | 65 | C3/C5 | 6 | Degenerate | ✓ |  |
| 266 | Surgical | 27 | L5/S1 | 4 | Non-degenerate | ✓ |  |
| 272 | Surgical | 26 | L5/S1 | 4.5 | Degenerate | ✓ |  |
| 276 | Surgical | 71 | C5/C6 | 6 | Degenerate | ✓ |  |
| 288 | Surgical | 73 | C6/C7 | 5 | Degenerate | ✓ |  |
| 292 | Surgical | 23 | L2/L3 | 4 | Non-degenerate | ✓ |  |
| 310 | Surgical | 37 | L4/L5 | 5 | Degenerate | ✓ |  |
| 319 | Surgical | 47 | L5/S1 | 5 | Degenerate | ✓ |  |
| 320 | Surgical | 68 | L4/L5 | 4 | Non-degenerate | ✓ | ✓ |
| 322 | Surgical | 48 | L4/L5 | 5 | Degenerate | ✓ | ✓ |
| 321 | Surgical | 22 | L4/L5 | 4 | Non-degenerate | ✓ |  |
| 328 | Surgical | 38 | L5/S1 | 6 | Degenerate | ✓ | ✓ |
| 329 | Surgical | 48 | L5/S1 | 5 | Degenerate | ✓ |  |
| 330 | Surgical | 32 | L5/S1 | 5.5 | Degenerate | ✓ |  |
| 332 | Surgical | 24 | L5/S1 | 10 | Degenerate | ✓ |  |
| 336 | Surgical | 40 | L5/S1 | 7 | Degenerate | ✓ | ✓ |
| 337 | Surgical | 24 | L5/S1 | 7 | Degenerate | ✓ |  |
| 338 | Surgical | 29 | L5/S1 | 8 | Degenerate | ✓ |  |
| 340 | Surgical | 42 | L5/S1 | 7 | Degenerate | ✓ |  |
| 342 | Surgical | 18 | L4/L5 | 6 | Degenerate | ✓ |  |
| 346 | Surgical | 46 | C5/C6 | 6 | Degenerate | ✓ |  |
| 348 | Surgical | 67 | C4/C5 | 9 | Degenerate | ✓ | ✓ |
| 350 | Surgical | 38 | L4/L5 | 9 | Degenerate | ✓ |  |
| 352 | Surgical | 82 | L4/L5 | 8 | Degenerate | ✓ |  |
| 355 | Surgical | 33 | L4/L5 | 5 | Degenerate | ✓ |  |
| 356 | Surgical | 26 | L5/S1 | 4 | Non-degenerate | ✓ |  |
| 357 | Surgical | 53 | L4/L5 | 4 | Non-degenerate | ✓ |  |
| 360 | Surgical | 50 | C6 | 5.5 | Degenerate | ✓ |  |
| 374 | Surgical | 37 | L4/L5 | 4 | Non-degenerate | ✓ |  |
| 375 | Surgical | 41 | L5/S1 | 6 | Degenerate | ✓ |  |
| 379 | Post mortem | 33 | L3/L4 | 5 | Degenerate | ✓ |  |
| 384 | Surgical | 47 | L5/S1 | 5 | Degenerate | ✓ |  |
| 388 | Surgical | 54 | C6/C7 | 6 | Degenerate | ✓ |  |
