# Supplementary material for: Hedgehog proteins and parathyroid hormone‐related protein are involved in intervertebral disc maturation, degeneration, and calcification
Source: JOR Spine. 2019 Nov 19;2(4):e1071. doi: 10.1002/jsp2.1071 (PMC6920702; doi:10.1002/jsp2.1071)
Supplement: Supplementary file 3 — Supporting information 3 PTHrP immunopositivity in young canine and human NP tissue [file JSP2-2-e1071-s003.docx]

**Supporting information 3. PTHrP immunopositivity in young canine and human NP tissue**


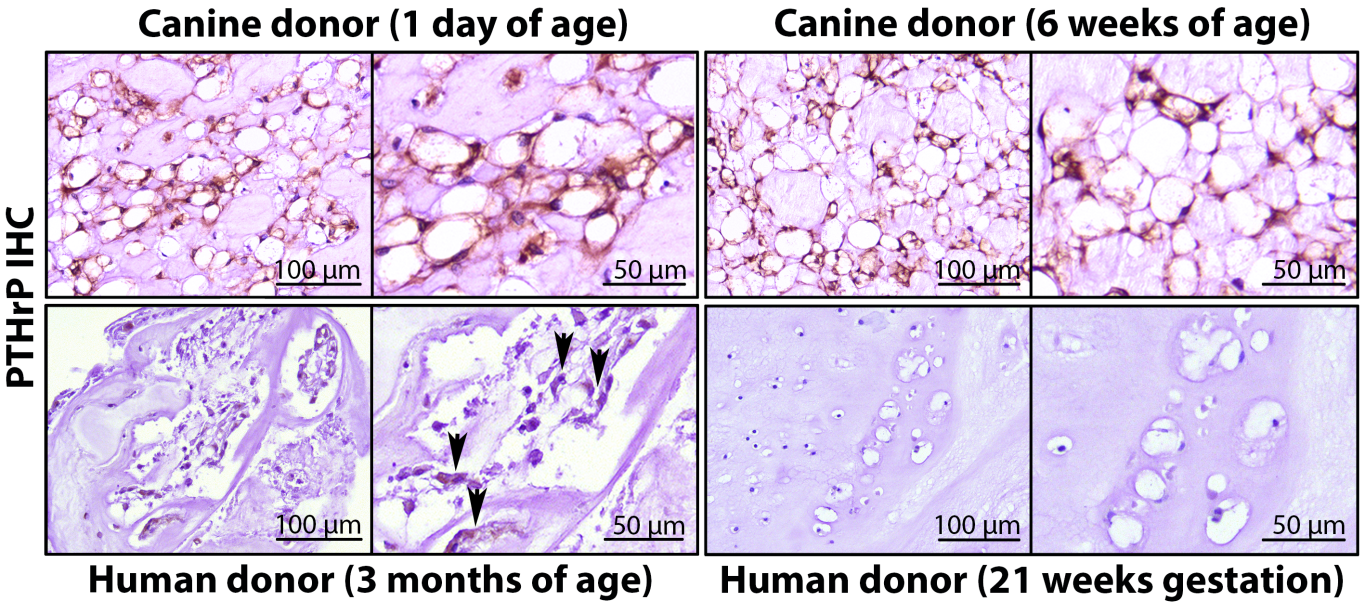


PTHrP: Parathyroid hormone-related protein. *n* = 8 (canine), 12 (human). Human NP tissue with PTHrP expression is shown in the lower left panel, whereas the lower right picture shows human NP tissue not expressing PTHrP. In every panel, the right picture is a two times magnified version of the left picture.
