# Supplementary material for: Hedgehog proteins and parathyroid hormone‐related protein are involved in intervertebral disc maturation, degeneration, and calcification
Source: JOR Spine. 2019 Nov 19;2(4):e1071. doi: 10.1002/jsp2.1071 (PMC6920702; doi:10.1002/jsp2.1071)
Supplement: Supplementary file 4 — Supporting information 4 Indian and Sonic hedgehog facilitate calcification in human and canine chondrocyte‐like cells in vitro. [file JSP2-2-e1071-s004.docx]

**Supporting information 4. Indian and Sonic hedgehog facilitate calcification in human and canine chondrocyte-like cells *in vitro*.**

**
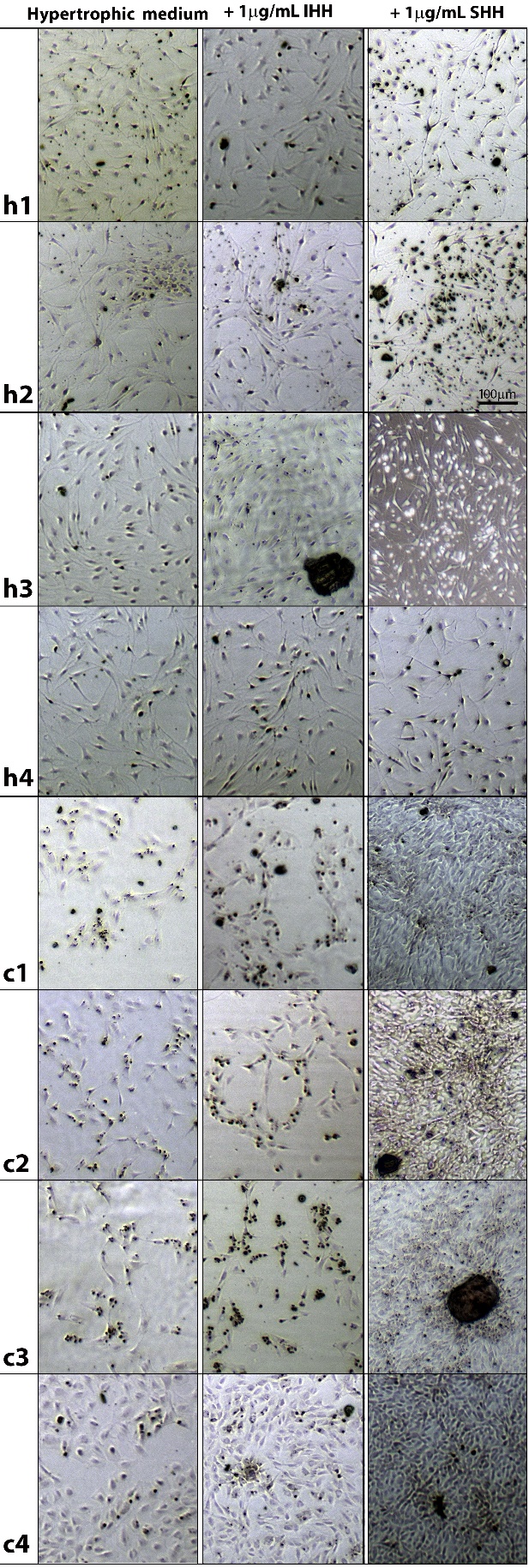
**

Alizarin Red S staining on 4 human (h1-h4) and 4 canine (c1-c4) CLC donors. CLC monolayers were treated with hypertrophic induction medium supplemented with/without 1 µg/mL IHH or SHH for 7 days. IHH: Indian hedgehog. SHH: Sonic hedgehog.
