# Supplementary material for: Hedgehog proteins and parathyroid hormone‐related protein are involved in intervertebral disc maturation, degeneration, and calcification
Source: JOR Spine. 2019 Nov 19;2(4):e1071. doi: 10.1002/jsp2.1071 (PMC6920702; doi:10.1002/jsp2.1071)
Supplement: Supplementary file 5 — Supporting information 5 PTHrP does not facilitate calcification in canine chondrocyte‐like cells in vitro. [file JSP2-2-e1071-s005.docx]

**Supporting information 5. PTHrP does not facilitate calcification in canine chondrocyte-like cells *in vitro*.**


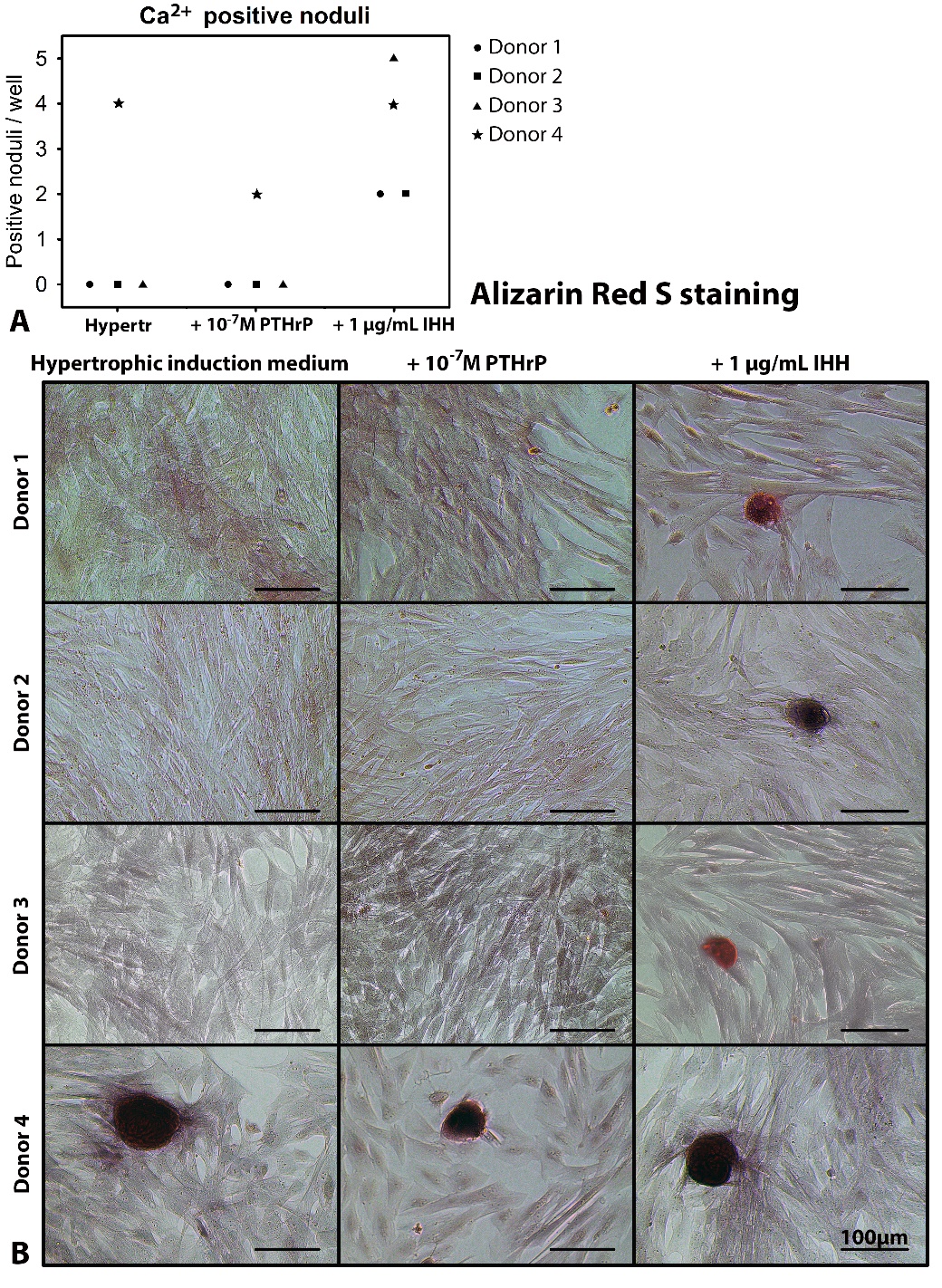


Alizarin Red S staining on 4 canine CLC donors. CLC monolayers were treated with hypertrophic induction medium supplemented with/without 10^-7^ M PTHrP (Parathyroid hormone related protein) for 7 days.
